# What is the prevalence of current alcohol dependence and how is it measured for Indigenous people in Australia, New Zealand, Canada and the United States of America? A systematic review

**DOI:** 10.1186/s13722-020-00205-7

**Published:** 2020-09-17

**Authors:** Teagan J. Weatherall, Katherine M. Conigrave, James H. Conigrave, K. S. Kylie Lee

**Affiliations:** 1 Faculty of Medicine and Health, Discipline of Addiction Medicine, NHMRC Centre of Research Excellence in Indigenous Health and Alcohol, The University of Sydney, King George V Building, 83-117 Missenden Road, Camperdown, NSW 2050 Australia; 2 Royal Prince Alfred Hospital, Drug Health Services, Camperdown, NSW Australia; 3 Centre for Alcohol Policy Research, La Trobe University, Bundoora, VIC Australia

**Keywords:** Indigenous, Australia, New Zealand, Canada, United States of America, Alcohol, Dependence, Prevalence, Screening, Assessment

## Abstract

**Background:**

Alcohol affects Indigenous communities globally that have been colonised. These effects are physical, psychological, financial and cultural. This systematic review aims to describe the prevalence of current (12-month) alcohol dependence in Indigenous Peoples in Australia, New Zealand, Canada and the United States of America, to identify how it is measured, and if tools have been validated in Indigenous communities. Such information can help inform estimates of likely treatment need.

**Methods:**

A systematic search of the literature was completed in six electronic databases for reports on current alcohol dependence (moderate to severe alcohol use disorder) published between 1 January 1989–9 July 2020. The following data were extracted: (1) the Indigenous population studied; country, (2) prevalence of dependence, (3) tools used to screen, assess or diagnose current dependence, (4) tools that have been validated in Indigenous populations to screen, assess or diagnose dependence, and (5) quality of the study, assessed using the Appraisal Tool for Cross-Sectional Studies.

**Results:**

A total of 11 studies met eligibility criteria. Eight were cross-sectional surveys, one cohort study, and two were validation studies. Nine studies reported on the prevalence of current (12-month) alcohol dependence, and the range varied widely (3.8–33.3% [all participants], 3–32.8% [males only], 1.3–7.6% [females only]). Eight different tools were used and none were Indigenous-specific. Two tools have been validated in Indigenous (Native American) populations.

**Conclusion:**

Few studies report on prevalence of current alcohol dependence in community or household samples of Indigenous populations in these four countries. Prevalence varies according to sampling method and site (for example, specific community versus national). Prior work has generally not used tools validated in Indigenous contexts. Collaborations with local Indigenous people may help in the development of culturally appropriate ways of measuring alcohol dependence, incorporating local customs and values. Tools used need to be validated in Indigenous communities, or Indigenous-specific tools developed, validated and used. Prevalence findings can inform health promotion and treatment needs, including funding for primary health care and specialist treatment services.

## Background

Alcohol affects Indigenous communities globally that have been colonised. These effects are physical, psychological, financial and cultural in nature [1, 2]. Colonisation, economic marginalisation, and governments’ systematic efforts to erode ‘Country’ (land, homelands) and culture causes trauma that continues to affect Indigenous Peoples. As a result, Indigenous Peoples are at increased risk of alcohol use disorders (AUDs), including alcohol dependence (moderate to severe AUD). Alcohol dependence can then erode the strengths of Indigenous Peoples—strong families, strong communities, strong culture, and traditional responsibilities. Concerns have been expressed about Indigenous Peoples’ lack of access to appropriate treatment for alcohol dependence [3, 4]. One step towards assessing likely treatment need is to have sound estimates of the prevalence of current alcohol dependence.

The World Health Organization’s (WHO) International Classification of Diseases (11th revision; ICD-11) and the American Psychiatric Association Diagnostic and Statistical Manual of Mental Disorders (5th revision; DSM-5) set out guidelines for identifying alcohol dependence or moderate to severe AUDs [5, 6]. In this paper we use the ICD-11 term ‘current alcohol dependence’. The World Health Organization describes dependence on alcohol as a strong internal drive to use alcohol that leads to inability to control use of alcohol, priority given to alcohol over other activities, and physiological features (e.g. ‘shakes’ when stopping use or first thing in the morning when waking up) [5]. However, it is not clear how well diagnostic guidelines for dependence apply to Indigenous Peoples [7].

Individuals who are dependent on alcohol typically experience greater harms than those with less severe AUDs, and require more intensive treatment [8, 9]. Globally, there is a need for reliable population data that can inform the planning of programs to prevent, identify and treat alcohol dependence [7, 10, 11]. Prevalence of current alcohol dependence (12-month) for the general population has been reported. For example, in Australia it is 1.4% [12], in New Zealand 1.3% (both using DSM-IV) [13], in Canada 4.1% (ICD-10) [14], and the United States of America (USA) 3.8% (DSM-IV) [15]. However, few studies assess the prevalence of current alcohol dependence in Indigenous communities in colonised countries (e.g. Australia, New Zealand, Canada and USA).

A range of tools have been used to screen for, assess or diagnose alcohol dependence. Screening tools include the Alcohol Use Disorders Identification Test (AUDIT) [16], CAGE [17], and the Michigan Alcoholism Screening Test (MAST) [18]. Diagnostic tools include the WHO-Composite International Diagnostic Interview (CIDI) [19], and The Schedule for Affective Disorders and Schizophrenia (SADS) [20]. Screening tools, if accurate, are useful to give an estimate of likely prevalence of alcohol dependence and so an estimate of likely treatment needs. Diagnostic tools, again if accurate, provide actual prevalence of current alcohol dependence and so a more precise estimate of likely treatment needs. However, the majority of tools have not been validated for Indigenous Peoples, and it is unclear how suitable they are for this context. For example, tools based on the DSM-V assess whether recurrent alcohol use affects obligations at work, school, or home [6]. This could mean attending school every day, pursuing further training or university, and going to work. But, in a remote Indigenous community in Australia, for example, there may not be a secondary school, nor university, and there may be limited employment opportunities [21]. Also, for some Indigenous people priority is given to community, culture and Country over ‘the home’.

To address these gaps in the literature, this systematic review aims to: 1) describe the prevalence of current (12-month) alcohol dependence in community or household samples in Indigenous Peoples in Australia, New Zealand, Canada and the USA; 2) identify which tools have been used to measure alcohol dependence for these peoples; and 3) identify if those screening or assessment tools have been validated, both for general and Indigenous populations.

## Methods

This systematic review has been registered with the International Prospective Register of Systematic Reviews (Prospero; ID number: CRD42019125352) [22].

A search of the literature was completed for studies published from 1 January 1989–9 July 2020. Searches were conducted of six electronic databases (Scopus, Medline, Embase, PsycInfo, CINAHL and Web of Science). We sought feedback on the search strategy (Table 1) from experts in drug and alcohol research, and a librarian.

**Table 1:** Search strategy used for systematic review

1	Indigenous OR Aborigin* OR “First Nation*” OR “First People*” OR “Torres Strait*” OR “Oceanic ancestry group*” OR Maori* OR “Native America*” OR “American Indian*” OR “Alaska* native*” OR “Native Canad*” OR Inuit* OR Metis*
2	Austral* OR “New Zealand*” OR Aotearoa* OR USA OR “United States*” OR Alaska* OR Canad* OR “North Americ*”
3	(substance w/3 disorder*) OR alcoholi* OR AUD OR (alcohol w/3 depend*) OR (alcohol w/3disorder*) OR (alcohol w/3 withdraw*) OR (alcohol w/3 tremor*) OR (alcohol w/3 shak*) OR (alcohol w/3 addict*)
4	Tool* OR Questionnaire* OR Survey* OR Instrument* OR Criteri* OR Valid* OR SDS OR “Severity of dependence*” OR CIDI OR “Composite International Diagnostic Interview*” OR “Indigenous Risk Impact Screen*” OR “Alcohol Use Disorders Identification Test*” OR DSM OR “Diagnostic and Statistical Manual*” OR ICD OR “International classification of disease*” OR CAGE OR MAST OR “Michigan Alcohol Screening Test*” OR DASS OR “Depression Anxiety Stress Scales*” OR “Alcohol Smoking and Substance Involvement Screening Test*” OR SADQ OR “Severity of Alcohol Dependence Questionnaire*” OR “Leeds Dependence Questionnaire*” OR ASI OR “Addiction Severity Index*”

**Fig. 1:**
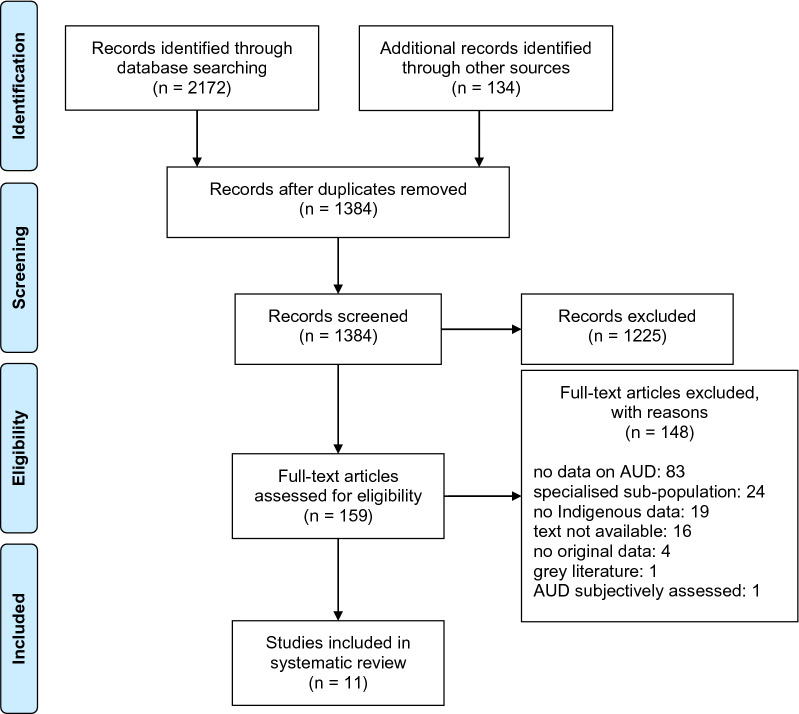
PRISMA diagram

As shown in the Preferred Reporting Items for Systematic Reviews and Meta-Analyses (PRISMA) diagram (Fig. 1), the search returned 2306 results, with 134 of these records identified through hand searching. Then 922 duplicates were removed. We screened titles and abstracts for the remaining 1384. Following title and abstract screen, 1225 were excluded. Of the remaining 159 studies, full-text screen was completed in duplicate by two researchers. Articles were excluded if (1) they did not report on prevalence of current alcohol dependence or on validation of tools, (2) where prevalence of dependence was assessed only in a specialised sub-population (such as patients engaged in AUD treatment or prison inmates, where dependence would be expected to be different from in the general population), (3) that did not report alcohol dependence data separately for Indigenous Peoples (from Australia, New Zealand, Canada or the United States), (4) where dependence was assessed subjectively (e.g. by clinical assessment, rather than using a tool), (5) full text not available, (6) grey literature, and (7) that did not present original data (i.e. commentary or review article). Eleven studies met criteria for inclusion in this systematic review (Fig. 1).

The following data were extracted from each included study: (1) Indigenous population(s) studied; country (or countries) of study (Australia, New Zealand, Canada, USA), (2) prevalence of current alcohol dependence in the Indigenous sample, (3) the tools used to screen, assess, or diagnose alcohol dependence, (4) the tools that have been validated in Indigenous populations to screen, assess, or diagnose alcohol dependence, and (5) the quality of the study, assessed using the Appraisal Tool for Cross-Sectional Studies (AXIS) [23]. Data were extracted in duplicate by two researchers. Differences were resolved by discussion, or where necessary by a third researcher.

## Results

The 11 included studies were of populations from the USA, Canada and New Zealand (USA = 8, USA/Canada = 2, New Zealand = 1) and were published from 1992 to 2020, inclusive (Table 2). Nine were prevalence studies, and two were validation studies. No studies were conducted in Australia.

**Table 2:** General characteristics of the studies identified (n = 11)

Authors (year)	Country	Year/s data collected	Indigenous people	Target population	Total Indigenous population size	Indigenous sample size: n	Study design
Prevalence studies (n = 9)							
Baxter et al. (2006) [32]	NZ	2003–2004	Maori	Maori adults nationally	Not reported	2595	Cross-sectional survey
Brave Heart et al. (2016) [21]	USA	2001–2002	Native American and Alaskan Natives (NA/AN)	USA adults for national census	5.2 million (in 2010)	701	Cross-sectional survey (census)
Gill et al. (1997) [24]	USA	Not reported	Native Americans	Native Americans living in Denver	Not reported	105	Cross-sectional survey
Grant et al. (2017) [31]	USA	2012–2013	Native Americans	USA adults	Not reported	Not reported	Cross-sectional survey (census)
Grant et al. (2004) [33]	USA	1991–1992	Native American and Alaskan Natives (NA/AN)	USA adults nationally	Not reported	Not reported	Cross-sectional survey
Kinzie et al. (1992) [25]	USA	1988 (versus 1969)	Native Americans	One village in western USA	426	131	Cross-sectional survey
Spicer et al. (2003) [28]	USA	1997–1999	Native Americans	Adults in Southwest and Northern Plains tribes; compared with data collected by the National Comorbidity Survey (NCS)	Not revealed^1^	A^2^. 1446 B^3^. 1638	Cross-sectional survey
Walls et al. (2020) [29]	USA and Canada	2017–2018	American Indian and First Nations Communities	Young adults from single Indigenous cultural group	Not reported	453	Cohort study
Whitbeck et al. (2006) [30]	USA and Canada	2002–2003	American Indian and First Nations Communities	Parents and caretakers from single Indigenous cultural group	Not reported	861	Cross-sectional survey
Validation studies (n = 2)							
Robin et al. (2004) [26]	USA	1989–1995	Native Americans	Southwestern and Plains adults (location not specified)	8578	A^4^. 456 B^5^. 214	Validation study
Saremi et al. (2001) [27]	USA	A^6^. 1991–1995 B^7^. 1991–1995 C^8^. 1992–1999	Native Americans	Location not specified	Not revealed^1^	A^6^. 307 B^7^. 275 C^8^. 2854	Validation study

^1^Withheld to protect the anonymity of the communities

^2^Southwest participants (Spicer et al.)

^3^Northern Plains participants (Spicer et al.)

^4^Southwestern participants (Robin et al.)

^5^Plains participants (Robin et al.)

^6^Psychiatric interview only (Saremi et al.)

^7^Both CAGE and psychiatric interview (Saremi et al.)

^8^CAGE questionnaire only (Saremi et al.)

### Demographics

The majority of studies (n = 7/11) were of one or two specific communities or tribal populations [24–30]. Four studies were national representative surveys [21, 31–33], of which two used census data [21, 31]. Of the seven local and regional prevalence studies, just one reported on the exact size of the total Indigenous population (426 persons) [25]. Of the two studies that used national census data, one did not report the total population size [31]; the other reported the total population of Native Americans as 5.2 million [21]. Where stated, sample sizes in prevalence studies ranged from 105 to 2595 persons [21, 24, 25, 28–30, 32], and in validation studies from 214 to 2854 [26, 27]. Two national studies did not report on the number of Indigenous people in their sample [31, 33]. The two reports that presented validation data drew on overlapping samples from a US Southwestern tribal population [26, 27]. Of studies confined to one or more regions, most did not describe rurality of participants. One US sample was from an urban region [24]; another US study reported that 38.2% of men and 35.9% of women were from a rural area [21].

Just under half the reports described the socioeconomic status of the sample. One US study reported that 67.8% of the sample were unemployed, 70.4% had graduated from high school and 11.4% had a university qualification [24]. In contrast, another US study reported that 43.6% of men and 50.3% of women were university educated [21]. In the latter sample, 42.4% of men and 70.1% of women had an individual income of less than $19,000 (USD). Lastly, one US study reported 54.3% of the sample as poor [28].

### Study design and recruitment

The majority of prevalence studies were cross-sectional surveys of community-based samples [24, 25, 28, 30, 32, 33], including the two studies that used US census data [21, 31]. One was a cohort study [29]. Five studies used stratified recruitment [28]; three specified this process was randomised [21, 31, 33], and one systematic [32]. Four studies used a convenience sampling strategy [24, 25], and two specified this was based on tribal rolls, attempting to contact all eligible individuals [29, 30].

Both of the validation studies [26, 27] used a range of recruitment methods, founded on pedigree-based sampling, and respondent-driven sampling.

### Prevalence of current alcohol dependence

In the nine studies that reported current (12-month) alcohol dependence for the whole sample (or this information could be calculated), the prevalence varied considerably (3.8–33.3%) [21, 24, 25, 28–33]. Six studies reported the prevalence stratified by gender [21, 25, 28–30, 33]. In all but one [30], the prevalence for males was higher than for females (range: 3.0–32.8% versus 1.3–7.6%). The studies which reported on specific communities or regions typically had higher prevalence (3.8–33.3%) [24, 25, 28–30], than those which had national samples (3.9–16.6%) [21, 31–33].

### Detecting alcohol dependence

All 11 studies used interviewer-administered tools to detect alcohol dependence. This included screening tools (CAGE-T, SADS-L, SMAST) and diagnostic tools (AUDADIS [Alcohol Use Disorder and Associated Disabilities Interview schedule] [34], CIDI, DIS [Diagnostic Interview Schedule] [35], UM-CIDI, WMH-CIDI) (Table 3). The CAGE-T is a variant of CAGE with an additional question (“Have you ever been treated for alcoholism?”) [27]. Three studies used tools that were administered by clinicians (e.g. psychologists, psychiatrists, or social workers) [25–27]; and two used tools administered by non-clinicians [21, 31]. Another four studies did not report who administered the tool(s) [24, 28, 32, 33]. Two studies used tools administered by Native community interviewers [29, 30]. One used an additional tool (SMAST) that was self-administered by some or all participants [26].

**Table 3:** Prevalence of current alcohol dependence and tools used in identified studies (n = 9)

Authors (year)	Recruitment strategy	Indigenous sample recruited			Current alcohol dependence			Interview/tool administration	Tool/s	Validated (y/n)	Validated in Indigenous communities (y/n)
		Male n (%)	Female n (%)	Age range (mean)^1^	Male n (%)	Female n (%)	Total n (%)				
Baxter et al. (2006) [32]	Stratified systematic	1048 (46.6%)	1547 (53.4%)	16-65+	Not reported	Not reported	3.9%	Interviewer-administered	WMH-CIDI (DSM-IV)	No	No
Brave Heart et al. (2016) [21]	Stratified random	314 (44.8%)	387 (55.2%)	18+	26 (8.4%) [1.84 SE]	17 (4.5%) [1.32 SE]	43 (6.1%)	Interviewer-administered (non-clinician)	AUDADIS-IV (DSM-IV)	Yes	No
Gill et al. (1997) [24]	Convenience	57 (54.3%)	48 (45.7%)	Not reported	Not reported	Not reported	35 (33.3%)	Interviewer-administered	DIS (DSM-III-R)	Yes	No
Grant et al. (2017) [31]	Stratified random	Not reported	Not reported	Not reported	Not reported	Not reported	16.6%	Interviewer-administered (non-clinician)	AUDADIS-V (DSM-IV)	Yes	No
Grant et al. (2004) [33]	Stratified random	Not reported	Not reported	18-65+	11.0%	7.4%	9.0%	Interviewer-administered	AUDADIS-IV (DSM-IV)	Yes	No
Kinzie et al. (1992) [25]	Convenience	Not reported	Not reported	Not reported	32.8%	6.2%	25 (18.8%)	Interviewer-administered (psychiatrists)	SADS-L (DSM-III-R)^2^	Yes	No
Spicer et al. (2003) [28]	Stratified	A^3^. 617 (43%) B^4^. 790 (48%)	A^3^. 829 (57%) B^4^. 848 (52%)	15-45 +	A^3^. 75 (12.2%) B^4^. 103 (13%)	A^3^. 11 (1.3%) B^4^. 64 (7.6%)	A^3^. 86 (6.0%) B^4^. 167 (10.2%)	Interviewer-administered	CIDI^5^ (DSM-III-R)	Yes	No
Walls et al. (2020) [29]	Convenience roll-based	42.3%	57.0%	24–27 (26.3)	6.8%	2.3%	4.2%	Native interviewer-administered	WMH-CIDI (DSM-IV-TR)	No	No
Whitbeck et al. (2006) [30]	Convenience roll-based	236 (27.4%)	625 (72.6%)	17-77 (41^6^; 39^7^)	3.0% [0.01 SE]	4.2% [0.01 SE]	3.8% [0.01 SE]	Native interviewer-administered	UM-CIDI (DSM-III-R)	Yes	No

^1^Mean presented where available

^2^Expanding questions on alcohol dependence and abuse and PTSD (Kinzie et al.)

^3^Southwest participants (Spicer et al.)

^4^Northern Plains participants (Spicer et al.)

^5^Alcohol-dependence questions only (Spicer et al.)

^6^Men

^7^Women

Of the eight tools used all but one have been validated for general population use [17–20, 34, 35] (Table 4). Only one tool used in the studies had not been validated (WMH-CIDI), but this tool has been shown to be consistent with clinical diagnoses [36]. Two screening tools, SMAST and CAGE/CAGE-T, were validated in overlapping Native American tribal samples [26, 27]. Both were compared against a lifetime diagnosis of dependence, according to SADS-L, rather than against current dependence. Area under the receiver operating characteristic (ROC) curve for the SMAST for men and women was 85–86% and 82–88%, respectively [26]. However, the authors conclude that the SMAST was not a valid tool in this setting because of elevated cut-offs, and big differences in cut-offs between populations and genders. Area under the ROC curve for CAGE for men and women was 81% and 75%, respectively [27] and for the modified CAGE-T was 79% and 76%, respectively [27].

**Table 4:** Validation studies in Indigenous communities of tools used to detect or assess dependence (n = 2)

Authors (year)	Recruitment strategy	Indigenous sample recruited			Interview/tool administration	Tool/s	Validated (y/n)	Validated in Indigenous communities (y/n)	Comment on validation
		Male n (%)	Female n (%)	Age range					
Robin et al. (2004) [26]	Other (selected from 3 multigenerational pedigrees)	A^1^. 205 (45%) B^2^. 101 (47%)	A^1^. 251 (55%) B^2^. 113 (53%)	21–50+	Interviewer-administered (clinical social worker and psychologist) SADS-L; interviewer-administered^1^ SMAST and self-administered^2^	SADS-L (DSM-III-R) SMAST (DSM-III-R)	Yes	SMAST (Native American population)	Authors suggest a cut-off of ≥ 5 for Southwestern men and women; ≥ 8 for Plains men and ≥ 6 for Plains women
Saremi et al. (2001) [27]	Other (selected from 3 multigenerational pedigrees)	A^3^. 157 (51%) B^4^. 96 (35%) C^5^. 1113 (39%)	A^3^. 150 (49%) B^4^. 179 (65%) C^5^. 1741 (61%)	21+	Interviewer-administered SADS-L (psychologist) and CAGE/CAGE-T	SADS-L and CAGE/CAGE-T (DSM-III-R)	Yes	CAGE (Native American population)	Authors suggest a cut-off score of ≥ 2

^1^Southwestern participants (Robin et al.)

^2^Plains participants (Robin et al.)

^3^Psychiatric interview only (Saremi et al.)

^4^Both CAGE/CAGE-T and psychiatric interview (Saremi et al.)

^5^CAGE questionnaire only (Saremi et al.)

### Study quality

Study quality was examined with the AXIS critical appraisal tool [23]. Aims and objectives were clear in ten of the 11 studies [21, 24–27, 29–33]. All studies were designed appropriately for their stated aims. The reference populations were clearly defined in all but one study [27], and the sample recruited was suitable for the study population for all but one study [27]. However, in four studies the sampling strategy was unlikely to represent the target population [24–27].

Methods were sufficiently described in eight studies to enable them to be repeated [21, 24, 27, 29–33] and eight studies described their basic data adequately [21, 24, 28–33]. Three studies described measures taken to address non-responders or missing data [29, 31, 32]. The results in all 11 studies were internally consistent. Only five studies mentioned funding sources or conflicts of interest [21, 27–29, 31] and three studies did not report on ethical approvals or consent [27, 32, 33].

## Discussion

To our knowledge this is the first review to examine the prevalence of current (12-month) alcohol dependence and to describe how it is measured in Indigenous Peoples in similarly colonised countries. We identified 11 reports published between 1992 and 2020 among Indigenous Peoples in New Zealand, Canada, and the USA. No reports from Australia were identified. We highlight the need for more and unbiased data on prevalence of current alcohol dependence in Indigenous communities. Also screening, assessment and diagnostic tools and instruments need to be validated for Indigenous Peoples in a cross-cultural context. Indigenous Peoples need to be consulted to see how alcohol dependence criteria are translated locally. Interviewers using mainstream tools and instruments need to understand Indigenous Peoples’ background and worldviews to understand alcohol dependence. Working in partnership with local Indigenous Peoples can inform this process. A clearer understanding of the prevalence of current alcohol dependence can help inform an estimate of the type and scope of alcohol intervention and treatment services needed for each community.

### Prevalence of current alcohol dependence

In all nine prevalence studies the total prevalence was similar or higher than in the general population (e.g. in the NZ study, 3.9% versus 1.3% [13]; USA only studies, 6.0–33.3% versus 3.8% [15]). As in general populations, in all but one study [30] males had a higher prevalence of dependence than females, where gender-specific prevalence was provided (3.0–32.8% versus 1.3–7.6%) [21, 25, 28, 29, 33].

Prevalence studies which used a stratified sampling strategy (n = 5) [21, 28, 31–33] tended to report a lower prevalence of dependence than those that used convenience sampling (n = 2) [24, 25] (3.9–16.6% versus 18.8–33.3%). However, studies using a combination of a convenience and roll-based strategy, contacting all eligible participants, reported a similarly low prevalence (3.8–4.2%) [29, 30] to studies with stratified sampling. Future studies need to strive for recruitment strategies that can yield a representative prevalence of alcohol dependence within communities. Strategies should include working with local Indigenous people, community leaders and service providers to better understand how to reach a broad range of individuals [37].

All but one [28] of the USA-only community samples, drawn from specific First Nations communities, [24, 25] had a higher prevalence of current alcohol dependence than US national surveys of First Nations peoples [21, 31, 33] (18.8–33.3% versus 6.1–16.6%). Because of selection bias applying to either individuals or whole communities the study findings may not be able to be generalised to other Indigenous communities.

### Detecting alcohol dependence

All but one tool used in the included studies have been validated for general communities. This review identified two tools used to screen for dependence (SMAST, CAGE/CAGE-T) that have been validated in Indigenous communities against criteria for lifetime dependence [26, 27]. These were found to have good accuracy. Nonetheless the authors questioned the validity of SMAST because of the need for high cut-offs, which vary by community and gender [26]. The authors concluded that the modified CAGE-T did not add any diagnostic value compared to the standard CAGE [27]. Future studies are needed to validate tools against the criteria of current alcohol dependence. This would better help inform current local prevention and treatment needs.

Some tools have been translated and validated in other languages (e.g. AUDIT, CIDI), but as complex as translation is, translating Indigenous values and worldviews (e.g. the passing on of oral lore, culture, family values, and traditional knowledge) can be a more demanding process [7]. As these values are not embedded in these tools, consultation is needed with Indigenous communities to determine how criteria of dependence could be best translated into local contexts [38]. Mainstream tools or dependence criteria may or may not be accurate when screening, assessing or diagnosing alcohol dependence in an Indigenous population. In some communities the differing context can make items used to assess dependence difficult to interpret, unless these are ‘translated’ through a cultural lens [39, 40]. For example, an Indigenous person’s responsibilities to community and to Country may be at least as important as responsibilities ‘in the home’. Furthermore, limited employment and educational opportunities in some communities may make questions about interference with work and study less relevant.

Some screening tools (e.g. AUDIT, CAGE) raise questions about feelings of guilt or shame in relation to drinking alcohol, for example, “How often during the last year have you had a feeling of guilt or remorse after drinking?” (AUDIT, Q7) [16]. If an individual indicates yes, the feeling of guilt may not necessarily relate to quantity or things said and done whilst drinking. Rather, the guilt could be from internalised racism, or internalised stigma of Indigenous people in relation to alcohol. On the other hand, an individual drinking heavily may experience no guilt or remorse if drinking large quantities is the social norm within that community. Interviewers utilising tools should consider an individual’s background and worldviews and as every Indigenous community is different, interviewers benefit from training by local Indigenous people [41].

None of the eight tools used to screen, assess or diagnose alcohol dependence were developed specifically for Indigenous Peoples. In an Australian context, the Indigenous Risk Impact Screen (IRIS) is an Indigenous-specific screening tool which has been validated in an Australian Indigenous population (Queensland). However, IRIS provides a combined measure of risk from alcohol and substance use. For this reason, studies using IRIS were not included in this systematic review.

### Implications for policy, practice and research

There is a need for more studies on prevalence of current alcohol dependence to inform prevention and treatment efforts in Indigenous communities internationally [42]. Assessing current prevalence is more useful than lifetime dependence for estimating current treatment needs. Large numbers of individuals who have experienced alcohol dependence may enter stable remission and not need treatment [29, 43]. In one national study on American Indians and Alaskan Natives, the prevalence of lifetime alcohol dependence was more than three times that of current dependence (19.7% versus 6.1%, respectively) [21]. Prevalence findings inform the need for health promotion and treatment strategies. The findings can inform the balance of funding allocation between primary health care and specialist treatment services. Health promotion and treatment needs may also vary by gender or age [44]. Accordingly, to assess need, studies should stratify results by gender and age.

Researchers and clinicians should work in partnership with local Indigenous community members to take into consideration local worldviews and culture [38, 41]. They also should ensure research approaches (e.g. sampling methods and approach to recruit individuals) are co-designed with Indigenous Peoples and the local community. This may require extensive consultation with Indigenous leaders and community members over a long period time to understand the community and people. Structured interviews in the hands of a culturally-aware interviewer could also assist screening, assessment and diagnostic tools to be understood in a cross-cultural context [39, 40, 45].

### Limitations

This systematic review excluded studies that examined only lifetime prevalence of alcohol dependence. A systematic review of that research would also be useful to indicate the prevalence of those who (if in remission) could relapse in the future. Another systematic review could focus on individuals with milder AUDs (harmful use or abuse). Studies of specialised sub-populations were excluded from the current study (e.g. hospital inpatients and prison inmates). The prevalence of alcohol dependence in these groups was expected to be higher than in representative community samples. It is possible that some eligible studies were overlooked, despite careful screening, because titles and abstracts did not give a clue to relevant data contained within. It is also possible that we may have missed studies that report on dependence in general populations, but have a sub-analysis reporting on Indigenous populations. Grey literature was excluded as were non-English language publications.

## Conclusion

Prevalence of current alcohol dependence varied considerably depending on study methods. Future studies should strive to recruit representative samples from Indigenous communities to give a more accurate estimate of the prevalence of current alcohol dependence. This review also highlighted the need for more alcohol dependence screening, assessment and diagnostic tools to be developed and validated by and for Indigenous populations. These tools should take into account local values and worldviews, and concepts underpinning the criteria in these tools should be clear. Leadership from local Indigenous community members, clinicians and researchers is a crucial part of future research in this area. Understanding the prevalence of current dependence can help inform communities. It can also help inform allocation of government funding for treatment needs.

## Data Availability

Data for this project is stored at the University of Sydney based at Drug Health Service, KGV Building, Missenden Road, Camperdown New South Wales, 2050 Australia.

## Abbreviations

AU: Australia
AUD: Alcohol use disorder
AUDADIS: Alcohol Use Disorder and Associated Disabilities Interview Schedule
AUDIT: Alcohol Use Disorders Identification Test
AXIS: Appraisal Tool for Cross-Sectional Studies
CIDI: WHO-Composite International Diagnostic Interview
DIS: Diagnostic Interview Schedule
DSM: Diagnostic and Statistical Manual of Mental Disorders
ICD: International Classification of Diseases
IRIS: Indigenous Risk Impact Screen
MAST: Michigan Alcoholism Screening Test
NZ: New Zealand
PRISMA: Preferred Reporting Items for Systematic Reviews and Meta-Analyses
ROC: Receiver operating characteristic
SADS: The Schedule for Affective Disorders and Schizophrenia
UM-CIDI: University of Michigan-Composite International Diagnostic Interview
USA: United States of America
WHO: World Health Organization
WMH-CIDI: World Mental Health-Composite International Diagnostic Interview
